# Colposcopic evaluation of cervix with persistent inflammatory Pap smear: A prospective analytical study

**DOI:** 10.4103/1742-6413.67112

**Published:** 2010-08-05

**Authors:** Papa Dasari, S Rajathi, Surendra V Kumar

**Affiliations:** Department of OBGY, JIPMER, Puducherry, India; 1 Department of Pathology, JIPMER, Puducherry, India

**Keywords:** Invasive carcinoma, non-specific inflammation, persistent inflammatory cellular changes, squamous intraepithelial lesion

## Abstract

**Background:**

Inflammatory Pap smear is the most common report received by a gynecologist. The cervical screening algorithm for benign cellular changes on the Pap smear recommends treatment of infection if indicated and a repeat Pap smear in 4 to 6 months time. If the inflammatory changes still persist, subject the patient to colposcopy. However, in practice, this is not followed, especially in developing countries like ours where proper screening protocols are not available. Hence, a good number of patients in the premalignant stage are being missed. This study was undertaken to evaluate patients with persistent inflammatory Pap smears without atypia using colposcopy.

**Methods:**

A prospective analytical study of 150 gynecologial patients with persistent inflammatory Pap smear between 2006 and 2008 in an out-patient setting. All of them were subjected to colposcopy and biopsy from the abnormal areas. The incidence of cervical intraepithelial neoplasia (CIN)/invasive carcinoma was calculated by proportions/percentages.

**Results:**

The incidence of invasive carcinoma was <1%. But, the incidence of pre-malignant lesions (CIN) was high (20.9%). CIN 2/3 and carcinoma in *situ* were present in 6.9% of the cases.

**Conclusions:**

Patients with persistent inflammatory Pap smears can harbour a high proportion of CIN and hence these patients will need further evaluation.

## INTRODUCTION

Chronic inflammation, either specific or non-specific, has been shown to be associated with malignancy and was thought to be one of the factors responsible for carcinogenesis. Persistent inflammation leads to increased cellular turnover, especially in the epithelium, and provides a selection pressure that results in the emergence of cells that are at a high risk for malignant transformation[1] Inflammatory Pap smear is the most common report the gynecologist receives even when the cervix appears normal. The original Papanicolau classification of class 2 smears denotes inflammation and the recommendation is to repeat the smear after treating the infection[2] However, this does not specify the type of infection, and the present reporting of Pap smear by the Bethesda system reports on specific infections and classifies it under benign cellular changes.[3] The cervical screening algorithm for benign cellular changes recommends treatment of infection if indicated and performing a repeat Pap smear in 4 to 6 months time and, if the inflammatory changes persist, to subject the patient to colposcopy.[4] In practice, however, this is not always followed, especially in developing countries. The significance of cervical cytology with atypia has been extensively studied. There is a great controversy regarding the optimal management of women with persistent inflammatory changes without atypia, some considering it less likely to be associated with dysplasia[5] and others recommending further evaluation as it is associated with a high incidence of cervical intraepithelial neoplasia (CIN).[6,7] Hence, we have undertaken this study with the following objectives: (1) to study the colposcopic features in the cervices of persistent inflammatory cellular changes on Pap smear (2) to study epithelial cell abnormalities by colposcopic biopsy of abnormal areas in such cases and (3) to determine the existence of significant cervical intraepithelial lesions or invasive carcinoma in patients with persistent inflammatory Pap smear.

## MATERIALS AND METHODS

This is a prospective analytical study conducted in the Department of Obstetrics and Gynecology between August 2006 and June 2008. One hundred and fifty women who showed persistent inflammatory changes on Pap smear were included in the study. Patients with persistent inflammatory changes with atypical or dysplastic cells, patients with Diabetes mellitus, pregnant women and patients with previous cervical surgery were excluded. The study was approved by the Institute Scientific and Ethical Committee.

Patients with a report of inflammatory Pap smear were selected at random for initial recruitment. These patients were advised to use Clotimazole or Betadine vaginal pessaries for a minimum of 6 days. Those with a clinical diagnosis of chronic pelvic inflammatory disease and showing inflammatory Pap smear were given Doxycycline and Metronidazole for a minimum period of 14 days along with vaginal pessaries. A repeat Pap smear was performed after a period of 2 weeks with Ayer's wooden spatula. No preparation of the cervix was undertaken at the time of sampling and women were not menstruating or using any vaginal douche or vaginal contraceptives at the time of sampling. If inflammatory cellular changes were reported again on the repeat Pap smear, these patients were subjected to colposcopic examination after taking informed consent.

The woman was kept in a dorsal position and the cervix was exposed by inserting a Cusco's speculum. Excess mucus was wiped off with a cotton swab soaked in saline. Five percent acetic acid was applied to the cervix and it was visualized using a binocular colposcope (OLYMPUS OSC 3FLA, M/s Olympus Optical Co. Ltd., Tokyo, Japan) under 40X magnification. Biopsies were taken from the abnormal areas (acetowhite areas and vascular abnormalities like fine punctuations, coarse punctuations, mosaic and areas showing atypical vasculature) and an endocervical curettage was performed. All the specimens were subjected to histopathological examination. The incidence of pre-malignant and malignant lesions was calculated as percentages.

## RESULTS

The clinical profile of the patients is shown in Table 1. The mean age was 37 years and the mean parity was 2.6. The most common symptom was vaginal discharge followed by pelvic pain and in 45% of the patients the clinical diagnosis was pelvic inflammatory disease. Abnormal uterine bleeding and erosion of the cervix also contributed to inflammatory smear in approximately 20% of the patients. The colopscopic features of patients with persistent inflammatory Pap smears are shown in Table 2. The most common feature was acetowhiteness (41.3%) followed by a combination of acetowhiteness and vascular abnormality (24.7%). Colposcopy was normal in nine patients and hence no biopsy was taken. Erosion was confirmed by colposcopy in 12% of the patients. Biopsy was also not performed when the margins of erosion were regular and these accounted for five cases. The correlation of inflammatory Pap smear with coloposcopic biopsy results is shown in Table 3. The most common biopsy result in patients with inflammatory Pap smear was chronic cervicitis (28.7%). Human papilloma virus (HPV) lesions accounted for 21.2% CIN 1 for 14.7% and CIN 2/3 for 4.4% of the cases. The two cases of carcinoma in *situ* and one case of invasive carcinoma also showed non-specific inflammation on Pap test. Ten percent (14/150) did not require biopsy.

**Table 1:** Clinical profile

*Clinical characteristic*	*Number*	*Percentage*
Mean age	37 years	
Parity	2.6	
Symptomatology		
White discharge per vaginum	67	44.7
Pelvic pain	41	27.3
AUB	18	12
Post-coital bleeding	9	6
Mass descending per vaginum	6	4
Post-menopausal bleeding	5	3.3
Dysmenorrhea	4	2.7
Clinical diagnosis		
PID	52	34.7
AUB	32	21.3
Cervix erosion	31	20.7
Unhealthy cervix	20	13.3
Uterovaginal prolapsed	6	4
Ovarian cyst	5	3.3
Fibroid uterus	4	2.7

AUB: abnormal uterine bleeding PID:Pelvic inflammatory disease

**Table 2:** Colposcopic features of patients with inflammatory Pap smear

*Inflammatory Pap smear*	*Normal*	*Erosion*	*Acetowhite areas*	*Vascular abnormalites*	*Acetowhite areas and vascular abnormalities*
Non-specific inflammation (*n* = 138)	7 (5)	18 (13)	58 (42)	24 (17.4)	32 (23.2)
Bacterial vaginosis (*n* = 6)	1	-	1	1	3
Candida albicans (*n* = 3)	1	-	1	-	1
Trichomonas vaginalis (*n* = 3)	-	-	2	-	1
Total (*n* = 150)	9 (6)	18 (12)	62 (41.33)	25 (16.7)	37 (24.7)

Figures in parenthesis are in percentage

**Table 3:** Correlation of inflammatory Pap smear with colposcopic biopsy

*Persistent inflammatory Pap smear (Cytodiagnosis)*	Histopathological diagnosis of colposcopic biopsy* (*n* = 136) (150-14)									
	*No biopsy*	*Normal tissue*	*SM*	*Chronic cervicitis (CC)*	*SM + CC*	*HPV lesions*	*CIN 1*	*CIN 2*	*Ca in *situ**	*Invasive Ca*
Non-specific inflammation (*n* = 138-12) (126)	12	17 (14)	2 (2)	35 (28)	17 (14)	29 (23)	18 (15)	5 (4)	2 (2)	1 (0.7)
Bacterial vaginosis (*n* = 6-1)	1	1		2			1	1		
Candida albicans (*n* = 3-1)	1	1								
Trichomonas vaginalis (*n* = 3)				1	1		1			
Total biopsies (150-14) (*n* = 136)	14	19 (13.9)	2 (1.5)	39 (28.7)	18 (13.2)	29 (21.2)	20 (14.7)	6 (4.4)	2 (1.5)	1 (0.7)

SM: Squamous metaplasia

* Percentages of pathological lesions calculated for 136 colposcopic biopsies, HPV: Human papilloma virus, Figures in parenthesis are in percentage

The correlation between clinical symptomatology and colposcopic features is shown in Table 4. The most common colposcopic feature is acetowhiteness followed by a combination of acetowhite areas and vascular abnormality, irrespective of the symptoms. Of the patients who presented with pelvic pain, 46% showed acetowhite areas and 24% showed a combination of acetowhite areas and vascular abnormality. This is slightly higher than the patients presenting with vaginal discharge per vaginum who showed acetowhite areas in 34% of the cases and a combination of acetowhite areas and vascular abnormality in 28% of the cases. Erosion was more common in patients with vaginal discharge than in those with pelvic pain.

**Table 4:** Correlation of clinical symptoms and colposcopic findings

*Clinical symptom*	*Colposcopic findings*					
	*Normal*	*Erosion*	*Acetowhite areas (AW)*	*Vascular abnormality (VA)*	*AW + VA*	*Total*
Vaginal discharge (*n* = 67)	3	13	23 (34)	9	19 (28)	67
Pelvic pain (*n* = 41)	3	3	19 (46)	6	10 (24)	41
AUB* (*n* = 18)	1	-	7	7	3	18
Post-coital bleeding (*n* = 9)	-	2	4	-	3	9
Mass per vaginum (*n* = 6)	-	-	3	2	1	6
Post-menopausal bleeding (*n* = 5)	-	-	3	1	1	5
Dysmennorrhea (*n* = 4)	2	-	2	-	-	4
Total (*n* = 150)	9 (6)	18 (12)	61 (40.7)	25 (16.7)	37 (24.7)	150

* AUB: abnormal uterine bleeding, Figures in parenthesis are in percentage

Table 5 shows the results of colposcopic biopsy in correlation with symptomatology. The patient with invasive carcinoma presented with pelvic pain and two patients with carcinoma in **situ** presented with post-coital bleeding. In patients who presented with vaginal discharge, the most common diagnosis was chronic cervicitis followed by HPV lesions in 17% and CIN 1 in another 17% of the cases. Patients with abnormal uterine bleeding also showed a significantly increased incidence of CIN (22.2%).

**Table 5:** Clinical symptoms and colposcopic biopsy results

*Clinical symptoms*	*Colposcopic biopsy histopathological examination results*									
	*No biopsy*	*Normal tissue*	*Squamous metaplasia (SM)*	*Chronic cervicitis (CC)*	*SM + CC*	*HPV lesions*	*CIN1*	*CIN2*	*CIN3*	*Invasion*
Vaginal discharge (*n* = 67)	8	12	1	17	3	12	12 (17.9)	2 (2.9)	-	-
Pelvic pain (*n* = 41)	3	5	-	9	11	8	3 (7.3)	1 (2.4)	-	1 (2.4)
AUB* (*n* = 18)	1	2	-	8	-	3	3 (16.7)	1 (5.6)	-	-
Post-coital bleeding (*n* = 9)	-	-	-	3	1	2	1	-	2 (22)	-
Mass per vaginum (*n* = 6)	-	-	-	1	2	3	-	-	-	-
Post-menopausal bleeding (*n* = 5)	-	-	-	1	1	-	1	2	-	-
Dysmennorrhea (*n* = 4)	2	-	1	-	-	1	-	-	-	-
Total (*n* = 150)	14 (9.3)	19 (12.7)	2 (1.3)	39 (26)	18 (12)	29 (19.3)	20 (13.3)	6 (4)	2 (1.3)	1 (0.7)

* AUB: abnormal uterine bleeding, Figures in parenthesis are in percentage

Of the benign lesions, chronic cervicitis and HPV changes were common. Acanthosis, koilocytosis, chronic cervicitis with koilocytosis, squamous metaplasia with koilocytosis, acanthosis with koilocytosis and koilocytic atypia were grouped under HPV changes. The incidence of invasive carcinoma was <1%. But, the incidence of pre-malignant lesions was high (20.9%). CIN 2/3 and carcinoma in **situ** together contributed to 6.9% of the cases.

## DISCUSSION

Cervical cancer screening was proved to be an important part of preventive health care of women. Attempts are being made to improve the efficacy of the screening programme to decrease the morbidity and mortality due to cervical cancer. The cervical screening algorithm for benign cellular changes on Pap smear recommends treatment of infection if indicated and a repeat Pap smear in 4 – 6 months time, and, if the inflammatory changes still persist, to subject the patient to colposcopy. However, in practice, this is not followed, especially in developing countries like ours, where proper screening protocols are not available/followed. Hence, a good number of patients in the pre-malignant stage are being missed. Most Obstetrician Gynecologists do not review the Pap smear result with the cytologists and 41% do nothing when inflammatory Pap smear is reported. Only 11% treat the infection and repeat Pap smear and 24% treat infection and do not repeat Pap smear.[8]

There are very few studies in the literature where the incidence of premalignant and malignant lesions was looked into in cases of inflammatory Pap smear. Inflammation can obscure few malignant cells and may result in high false negative rates and the same may be reduced by employing liquid based cytology.[8] However, it was reported that liquid based cytology was not cost-effective for developing countries and the recent studies did not report a statistically significant difference of accuracy between conventional Pap test and liquid based cytology.[9] The main reason for false-negative reports of cytology were found to be sampling errors, with sampling errors as high as 42.5% being suboptimal and 17.5% being inadequate for interpretation.[10] Mc Lachlan and colleagues studied the colposcopic features and biopsy results of 102 women with persistent inflammatory Pap smears and found 19% cases of CIN 2 or worse.[11] This is almost similar to the present study.

The mean age and parity in the present study was higher (37 years and 2.6) than that of Seckin and colleagues, where the mean age was 30.2 years and the parity was 1.7. The most common persistent inflammatory Pap smear subjected to colposcopy was presumed to be non-specific in the study of Seckin and collegues, as they did not specify on the type of inflammatory smear.[6] In the present study, 92% were non-specific inflammation and only 8% were specific inflammation due to *Trichomonas vaginalis, Candida albicans* and bacterial vaginosis. Wilson and colleagues included bacteriological cultures of cervicovaginal smears to diagnose specific infections and found an increased incidence of sexually transmitted infections in patients <25 years of age and in many showing abnormal colposcopic features in this age group^7^. Colposcopy was normal in 9.3% of the patients in the present study, which was much lower than that of Seckin and collegues, who reported 29.1% to be normal. A very high percentage (62.5%) of normal colposcopic findings was reported by Wilson and colleagues in 96 patients of inflammatory Pap smears. This is in contrast with the results of the present study.

Colposcopic biopsy showed benign lesions in 48.2% of the cases in the study done by Seckin and collegues, which was lower than that reported in the present study. Seckin reported a very high incidence of HPV-related lesions (64.5%) where as Frisch reported an incidence of only 8%.[12] HPV related lesions constituted only 19.4% in the present study. The incidence of pre-malignant lesions in the present study (20.9%) was closer to that of Frisch (23.5%) but much higher than that of Seckin and colleagues (8% - 18 out of 224). There were no cases of malignancy in the series of Seckin and Frisch but in the present study, one case was found. Recently, Hammes and colleagues evaluated the population of macrophages during the cervical malignant transformation and its influence on CIN in cervical biopsy specimens. They concluded that macrophage count and inflammation increased linearly with disease progression. Inflammation was present in 25%, 46.1%, 58.4% and 89.3% of normal, Low grade squamous intraepithelial lesion (LGSIL), (High grade squamous intraepithelial lesion) HGSIL and squamous cell carcinoma, respectively.[13]

Seckin recommends colposcopic evaluation of patients with persistent inflammatory Pap smear despite therapy in any population in any part of the World. Frisch is of the opinion that colposcopy of women with cytologic diagnosis of inflammatory epithelial changes may be a useful way to detect otherwise unrecognized cases of CIN, and the present study highlights these statements.

## CONCLUSIONS

Patients with persistent inflammatory Pap smears can harbour a high proportion of CIN and HPV infection and hence these patients will need further evaluation by colposcopy.

## COMPETING INTEREST STATEMENT BY ALL AUTHORS

No competing interest to declare by any of the authors.

## AUTHORSHIP STATEMENT BY ALL AUTHORS

Each author acknowledges that this final version was read and approved. All authors of this article declare that we qualify for authorship as defined by ICMJE http://www.icmje.org/#author. Each author has participated sufficiently in the work and take public responsibility for appropriate portions of the content of this article.

## ETHICS STATEMENT BY ALL AUTHORS

This study was conducted with approval from Institutional Review Board (IRB) (or its equivalent) of all the institutions associated with this study. Authors take responsibility to maintain relevant documentation in this respect.

## EDITORIAL / PEER-REVIEW STATEMENT

To ensure integrity and highest quality of CytoJournal publications, the review process of this manuscript was conducted under a double blind model(authors are blinded for reviewers and reviewers are blinded for authors) through automatic online system.
